# Neoadjuvant chemotherapy in breast cancer: early response prediction with quantitative MR imaging and spectroscopy

**DOI:** 10.1038/sj.bjc.6602948

**Published:** 2006-02-07

**Authors:** D J Manton, A Chaturvedi, A Hubbard, M J Lind, M Lowry, A Maraveyas, M D Pickles, D J Tozer, L W Turnbull

**Affiliations:** 1 The Postgraduate Medical Institute of the University of Hull Division of Cancer in association with the Hull York Medical School, The University of Hull, Hull, East Yorkshire HU6 7RX, UK; 2 The Department of Clinical Oncology, Hull and East Yorkshire Hospitals NHS Trust, The Princess Royal Hospital, Saltshouse Road, Hull, East Yorkshire HU8 9HE, UK; 3 The Breast Screening Unit, Hull and East Yorkshire Hospitals NHS Trust, Castle Hill Hospital, Castle Road, Cottingham, East Yorkshire HU16 5JQ, UK; 4 NMR Research Unit, Institute of Neurology, Queen Square, London WC1N 3BG, UK

**Keywords:** breast neoplasms, chemotherapy, magnetic resonance, MR, spectroscopy, contrast enhancement, diffusion study

## Abstract

A prospective study was undertaken in women undergoing neoadjuvant chemotherapy for locally advanced breast cancer in order to determine the ability of quantitative magnetic resonance imaging (MRI) and proton spectroscopy (MRS) to predict ultimate tumour response (percentage decrease in volume) or to detect early response. Magnetic resonance imaging and MRS were carried out before treatment and after the second of six treatment cycles. Pharmacokinetic parameters were derived from *T*_1_-weighted dynamic contrast-enhanced MRI, water apparent diffusion coefficient (ADC) was measured, and tissue water : fat peak area ratios and water *T*_2_ were measured using unsuppressed one-dimensional proton spectroscopic imaging (30 and 135 ms echo times). Pharmacokinetic parameters and ADC did not detect early response; however, early changes in water : fat ratios and water *T*_2_ (after cycle two) demonstrated substantial prognostic efficacy. Larger decreases in water *T*_2_ accurately predicted final volume response in 69% of cases (11/16) while maintaining 100% specificity and positive predictive value. Small/absent decreases in water : fat ratios accurately predicted final volume non-response in 50% of cases (3/6) while maintaining 100% sensitivity and negative predictive value. This level of accuracy might permit clinical application where early, accurate prediction of non-response would permit an early change to second-line treatment, thus sparing patients unnecessary toxicity, psychological morbidity and delay of initiation of effective treatment.

Recent statistics confirm that breast cancer remains one of the most prevalent and serious forms of neoplastic disease in the UK. Breast cancer accounts for more than one in four female cancer cases and currently demonstrates age-standardised incidence and mortality rates of 117 and 31 per 100 000, respectively (Toms, 2003).

Some primary breast cancers are considered inoperable at diagnosis owing to their problematical location and size, with tumours greater than 3 cm in diameter being associated with an increased risk of disseminated disease. In such cases, neoadjuvant chemotherapy is routinely used before surgery to increase the chances of a successful outcome. Assessing tumour response to chemotherapy is crucial to patient management and is currently achieved by monitoring changes in tumour size using clinical examination backed up by longitudinal X-ray or ultrasound mammography. A poor response to the primary treatment regime usually prompts either change of chemotherapy regime or an early resort to surgery. Poor response might also require a greater degree of surgical intervention.

Repeated X-ray mammography (XRM) has several drawbacks: discomfort, radiation exposure, geometric distortion owing to compression and magnification render tumour volume measurements less accurate than magnetic resonance imaging (MRI), and tumour may be impossible to distinguish from dense glandular tissue and fibrosis. Magnetic resonance imaging has been shown to correspond better with pathological size measurement than XRM (Esserman *et al*, 1999; Drew *et al*, 2001) and to be less obscured by dense breast diseases. Ultrasound measurement is more accurate than XRM, but fails when the tumour to be measured is larger than the field of view or complex in shape. Magnetic resonance imaging is also more accurate than ultrasound at detecting small volume residual tumour.

However, even if tumour volume changes can be accurately assessed by MRI, they may manifest themselves later than changes in underlying tumour function such as vascular density or permeability (Wasser *et al*, 2003). Therefore, vascular or metabolic parameters might provide a more sensitive indicator of early tumour response, thus permitting individual treatment regimes to be adjusted more rapidly, and sparing patients unnecessary morbidity, expense and delay in initiation of effective treatment. Treatment-induced changes in metabolism can be investigated using both positron emission tomography (Byrne *et al*, 2004; Kumar and Alavi, 2004; Weber, 2005) and single photon emission computed tomography (Buscombe *et al*, 1997), but the clinical utility of such examinations is reduced by the need to limit repeated radiation doses.

Treatment-induced changes in tumour neovasculature can be assessed using dynamic contrast-enhanced MRI (DCE-MRI) and quantified using pharmacokinetic (PK) modelling (Padhani, 2002; Knopp *et al*, 2003). Diffusion-weighted MRI can be used to detect changes in the apparent diffusion coefficient (ADC) for tissue water associated with changes in tissue and intracellular structure (Zhao *et al*, 1996; Gallons *et al*, 1999; Ross *et al*, 2003; Moffat *et al*, 2005). Proton magnetic resonance spectroscopy (MRS) can be used to characterise breast lesions through differences in the ratio of fat and water signals (Chu *et al*, 1987; Sijens *et al*, 1988) or the intensity of signal from choline-containing compounds (Bradamante *et al*, 1988; Katz-Brull *et al*, 2002; Bolan *et al*, 2003; Jacobs *et al*, 2004; Jacobs *et al*, 2005). Both fat : water ratios (Jagannathan *et al*, 1998) and choline levels (Preul *et al*, 2000; Jagannathan *et al*, 2001; Schwarz *et al*, 2002; Meisamy *et al*, 2004) have also been used to monitor treatment-induced changes, and phosphorous MRS has been used to predict response using differences in the levels of phosphocholine or phosphomonoesters (Shukla-Dave *et al*, 2002a, 2002b; Arias-Mendoza *et al*, 2004). Sodium MRI might also be useful in monitoring treatment response (Kline *et al*, 2000; Schepkin *et al*, 2005).

A prospective study was, therefore, undertaken whereby three of these techniques (DCE-MRI, ADC measurement and MRS fat : water ratios) were carried out in the same patients to determine in each technique the relative prognostic utility. The study hypothesis was that a combination of the quantitative data from the three techniques would lead to a synergistic increase in prognostic accuracy and, therefore, provide an accurate, reliable and non-invasive early indication of tumour response to chemotherapy, which could potentially have a positive influence on patient management.

## MATERIALS AND METHODS

### Chemotherapy regime and relative timing of MRI

All women who were scheduled to undergo neoadjuvant chemotherapy for primary inoperable locally advanced breast cancer were approached to join the study, which had received Local Research Ethics Committee approval (reference number 03/00/038). Longitudinal MRI was carried out using a 1.5 T Signa Advantage clinical MRI scanner (International General Electric, Milwaukee, WI, USA) using a dedicated bilateral breast coil (Machnet BV, Eelde, Netherlands). A standard dosage chemotherapy regime was used involving intravenous administration of epirubicin (bolus: 60 mg m^−2^ body surface area), cyclophosphamide (bolus: 600 mg m^−2^) and 5-fluorouracil (continuous infusion by pump: 200 mg m^−2^ per day). Good clinical practice, including checking patients' cardiac status before therapy if necessary, was adhered to throughout the study. Magnetic resonance imaging and MRS were carried out at three time points: before the first course of chemotherapy (TP_0_), after the second course but no more than 55 days after the first course (TP_2_) and after the final (generally sixth) course (TP_F_). Therapeutic response was confirmed after surgery by pathological examination.

### MR imaging and spectroscopy

All the MRI data at each time point were acquired in a single imaging session with an average length of 1 h. Dynamic contrast-enhanced MRI was carried out first, during which gadopentetate dimeglumine (formerly known as Gd-DTPA; Schering Health Care, Burgess Hill, UK) was injected as a bolus at a dose of 0.1 mmol kg^−1^ body weight, using a two-dimensional, *T*_1_-weighted multislice, fast RF spoiled gradient echo (FSPGR) sequence. In order to attain adequate tissue coverage in all cases, imaging was carried out in either the coronal (30 cm field-of-view, FOV) or sagittal (20 cm FOV) planes (256 × 128 matrix in all cases) with five, seven or nine slices (4–9 mm thick, 2 mm gaps). Scan timing parameters, therefore, ranged as follows: TR=8.8–11.1 ms; TE=4.2 ms fractional; flip=30°; temporal resolution=10.5–14.5 s; 35 time points per slice; scan time=6 min 8 s to 8 min 28 s. Dynamic contrast-enhanced MRI was preceded by proton density-weighted FSPGR imaging (TR/TE/flip=120 ms/4.2 ms fractional/8°) to enable correction for differences in native tissue *T*_1_.

A single region-of-interest (ROI) that best delimited the lesion present was drawn, by an experienced radiologist, for each DCE-MRI study. In-house software (developed using the IDL language, Research Systems Inc., Boulder, CO, USA) was then used to measure the mean pixel signal intensity (SI) within the ROI for the proton density image (SI_PD_) and each of the images in the *T*_1_-weighted series (
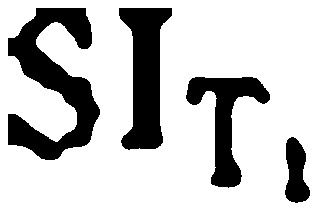
). These data were then used to calculate an enhancement factor (EF) time series proportional to the concentration of gadopentetate dimeglumine, with differences/changes in the native tissue *T*_1_ between patients/visits being corrected for using the proton density SI and the mean pre-contrast *T*_1_ SI as obtained from a user-defined number of baseline points (SI_0_) (Hittmair *et al*, 1994). The equations for EF calculation were 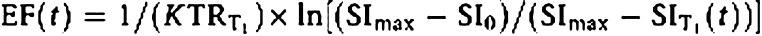 and 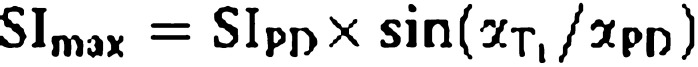, where 
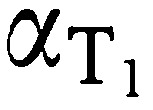
 and 
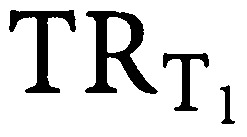
 are the flip angle and TR for the dynamic *T*_1_-weighted series and *α*_PD_ is the flip angle for the proton density-weighted image. A value of 11.04 was used for the constant *K*, this having been determined through calibration experiments using gels of known *T*_1_ values and being valid for 
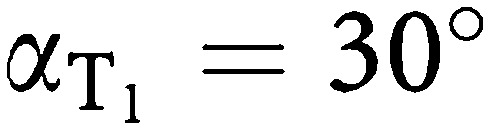
 only.

A two-compartment PK model (Buckley *et al*, 1994; Tofts, 1997) was used to calculate the amplitude, or initial slope, of the EF time series (this being proportional to the microvessel transfer constant *K*^trans^ (Tofts, 1997), itself proportional to the permeability surface area product) and the rate constant (*K*_ep_). The model equation was 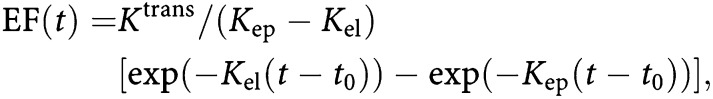 where *K*_el_ represents contrast clearance from the plasma and *t*_0_ represents the variable arrival time of the contrast agent. The extracellular–extravascular tissue volume fraction (*V*_e_) was also estimated using the *K*^trans^/*K*_ep_ ratio (assuming equal influx and efflux vascular permeability; Tofts, 1997). Curve fitting was carried out (using mean ROI data, not pixel-by-pixel) using a nonlinear least-squares algorithm implemented within the in-house, IDL software, and the model-independent maximum enhancement factor (MEF) was also recorded.

Tumour volume was measured using manually traced ROIs drawn on high-resolution three-dimensional, post-contrast, fat-suppressed FSPGR images. Again, sequence acquisition parameters were varied in order to obtain adequate tissue coverage in all cases and these ranged as follows: TR/TE/flip=14.6/4.2 ms fractional/30°; plane/FOV/matrix=sagittal/18–24 cm/512 × 256; slice number/thickness=25/2.5–5 mm. Tumour volumes were measured, rather than more typical linear measurements, as this was considered to be the most rigorous and accurate, although most time-consuming, way of quantifying lesion size. Tumour volume has been shown to correlate more closely than tumour diameter with disease-free survival in breast cancer patients and it may also provide a more sensitive characterisation of tumour response than one-dimensional measurements (Partridge *et al*, 2005).

It was assumed that neoplastic tissue would not demonstrate substantial ADC anisotropy; therefore, in an attempt to keep scan time to a minimum, ADC was measured in the frequency-encode direction only (left-to-right), which was known to demonstrate the best imaging homogeneity and stability. The Stejskal–Tanner technique (Stejskal and Tanner, 1965) was implemented using a single fat suppressed slice (axial, 22 cm FOV, 128^2^ matrix, 7 mm thick) single-shot, spin-echo echo-planar imaging and with the following diffusion weightings: 0, 14, 55, 125, 222, 346, 499 and 680 s mm^−2^. The ADC values were calculated on a whole ROI basis (Gibbs *et al*, 2001), which included a correction for the bias inherent in magnitude image reconstruction at low signal-to-noise ratios (especially true for higher *b*-values) (Miller and Joseph, 1993).

**Figure 1:**
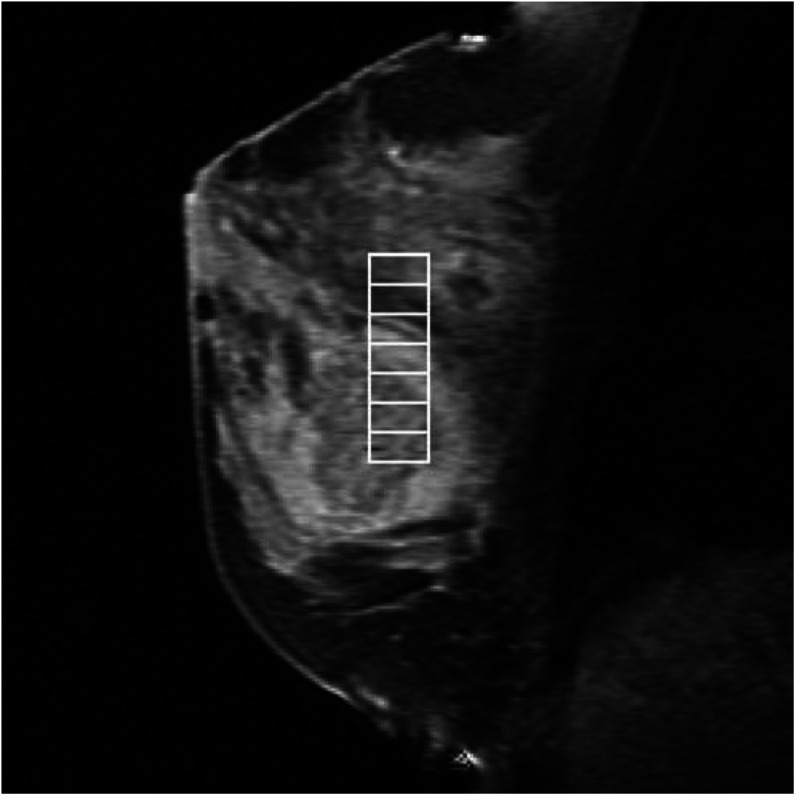
An example of the seven-voxel spectroscopic imaging localisation technique in a pre-chemotherapy tumour. (The bottom two voxels can be seen to contain wholly tumour, and spectral analysis would have been limited to these voxels.) The image on which the voxels are overlaid is the single slice, *T*_1_-weighted, sagittal localiser scan acquired with fat suppression post-contrast and immediately before spectroscopy.

The relative proportions of spectroscopic signal arising from tissue water and lipids were measured at echo times of 30 and 135 ms using a one-dimensional stimulated echo (STEAM) spectroscopic imaging sequence (without chemical shift selective suppression of any moieties) and a repetition time of 3 s. Thirty-two phase-encode steps were used over a 16 cm superior–inferior FOV with STEAM excitation limited to seven adjacent voxels (1.0 × 0.5 cm in the anterior–posterior and left–right directions, respectively, giving a nominal voxel volume of 0.25 ml). Figure 1 shows the seven spectroscopic voxels in a representative tumour before chemotherapy. To ensure that the same region of tissue was examined in follow-up studies, MRS voxels were relocated, by an experienced radiologist, after careful comparison of the breast architecture surrounding the lesion with the architecture demonstrated on hard copies of previous voxel locations. Changes in tumour choline were not measured, as the predictive utility of the technique had not been established in the literature at the time of study design.

Spectral analysis was carried out using the SAGE IDL package (International General Electric, Milwaukee, WI, USA). In order to decrease the degree of inter-voxel signal contamination, mild spatial apodisation was applied (Fermi filter: diameter/width=90%/5%), which resulted in an effective voxel volume of 0.33 ml (Jackson *et al*, 1994). To minimise partial volume contamination from adipose tissues outside the tumours, only signals from those voxels contained wholly within the tumour were averaged; then, water and fat signal intensities were measured by frequency-domain fitting of the water peak (4.7 p.p.m.) and the dominant lipid peaks at 1.3 and 0.9 p.p.m. (representing the methylene –(CH_2_)_*n*_– and terminal methyl –CH_3_ lipid moieties, respectively). Spectroscopic water content (%*W*_MRS_) was then quantified using the ratio of the water signal to that of the sum of water and the two dominant fat signals. Water *T*_2_ was also estimated using a crude two-point method: *T*_2_∼(TE_135_−TE_30_)/ln(area_30_/area_135_).

### Biopsy data

Patients' notes were reviewed to collate data obtained from histopathological analysis of specimens acquired via pre-chemotherapy core biopsy and final surgery. Parameters of interest were histological tumour subtype, tumour grade (pre-chemotherapy), the presence or absence of ductal carcinoma *in situ* (DCIS) and pre-chemotherapy oestrogen and progesterone receptor status (ERS and PRS) scores. Oestrogen and progesterone receptor status scores were recorded in the notes on either a six- or eight-point scale and converted to continuous data by calculating the ratio of the score to maximum possible score. The maximum diameter of the resected tumour, as calculated by the pathologist, was also collated and compared to the maximum diameter recorded in the final clinical MRI report using the Limits of Agreement method proposed by Bland and Altman (1986).

### Quantifying and correlating parameter changes and tumour response

Changes in parameter values between TP_0_ and TP_2_ were calculated either as absolute differences, 
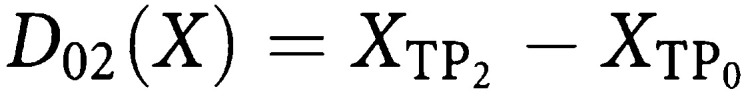
, or percentage change, 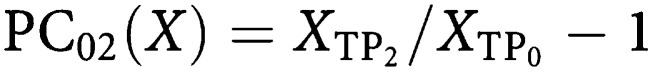, as deemed most appropriate. A change in tumour volume at the end of treatment, PC_0F_(*V*), exceeding −65% was taken as the cutoff between partial response (PR) and stable disease (SD), this being equivalent to a decrease in cross-sectional area of 50%, itself broadly equivalent to the RECIST criterion of a 50% decrease in the product of maximum orthogonal diameters (Therasse *et al*, 2000). No attempt was made to predict final tumour size in this study, as this is not likely to alter patient management before surgery.

All statistical analyses were carried out using the SPSS package (SPSS Inc., Chicago, IL, USA). Correlation between *D*_02_(*X*), PC_02_(*X*) or continuous/ranked pathology data and PC_0F_(*V*) was assessed using the Spearman non-parametric test (two-tailed). Correlation between dichotomous pathology data (presence/absence of DCIS) and categorical pathology data (cancer type) was assessed using the Mann–Whitney and Kruskal–Wallis non-parametric tests (two-tailed), respectively. Prognostic efficacy was assessed using receiver–operator characteristic (ROC) curves (Altman and Bland, 1994), with PR as the positive result, and the areas contained underneath them (AUC) calculated.

Variables were combined, in the hope of attaining a synergistic increase in prognostic efficacy, using logistic regression analysis (LRA) modelling, a statistical technique that maximises binary classification accuracy using a linear combination of weighted input variables plus a constant term (Hosmer and Lemeshow, 1989). Backwards conditional elimination of input variables (*P*>0.10) was used in order to prevent over-parameterisation of models, and ordinal variables were treated as categorical with the most benign category used as the indicator reference. Some statistical confidence intervals (CIs) were obtained using tabulated values (Anonymous, 1982).

## RESULTS

### Patient recruitment, withdrawal and other excluding factors

A total of 46 women were recruited into the study over a period of approximately 2 years; however, six (13%) of these subsequently withdrew. One case of atypical, inflammatory lobular cancer was excluded from data analysis on the grounds that it is impossible to measure tumour volume accurately in such tumours. One case in which histology only revealed DCIS was also excluded on the grounds that DCIS is too dissimilar to the other tumour types present, and chemotherapy is not the treatment of choice for it (2/40=5%).

**Figure 2:**
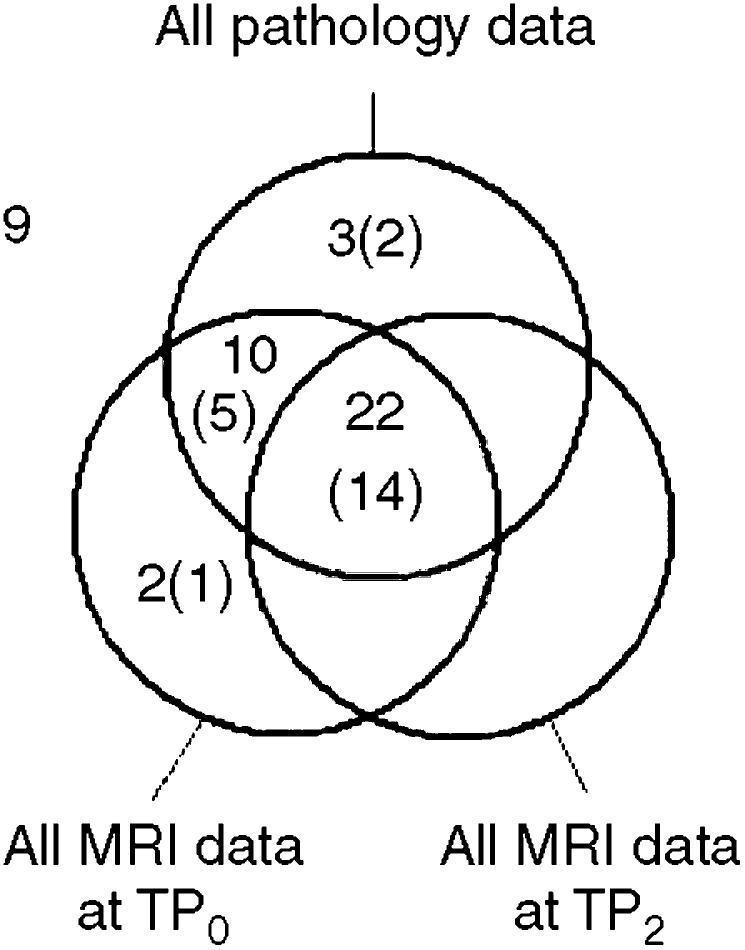
A Venn diagram showing the availability of valid data for the 46 women recruited into this study (with nine cases having to be excluded completely). Numbers in parentheses indicate the number of cases where DCIS was present.

In three of the remaining 38 cases, the second MRI examination was severely delayed owing to clinical complications and was carried out after the chosen cutoff of 55 days, thus necessitating the exclusion of the TP_2_ (but not the TP_0_) data. In addition, it was not possible to acquire ADC and MRS data on every occasion because of a number of problems including hardware failure and an inability to acquire sufficient signal to permit sequence optimisation. Such failures occurred during the TP_0_ scan, necessitating complete exclusion of the patient from data analysis, in four cases, and at TP_2_, necessitating removal of TP_2_ data only, in nine cases. A total of 13 failures in the 73 examinations (38 at TP_0_ and 35 at TP_2_) represent a failure rate of 18%. Results will, therefore, be presented for 22 cases for which both TP_0_ and TP_2_ data were available and an additional 12 cases for which data were available at TP_0_ only (see Figure 2).

Neither tumour volume measurements nor PK analysis were affected at any time point, as these were based on three-dimensional and DCE-MRI images acquired before ADC and MRS were attempted. Therefore, tumour volume response, PC_0F_(*V*), could be quantified in all cases. Pharmacokinetic data and tumour volume data calculated in cases where ADC and MRS data were not acquired were voluntarily excluded, so as not to bias statistical power in favour of PK/volume parameters. In the 22 cases for which valid TP_2_ data were available, 18 (82%) of the women had received two courses of chemotherapy at the time of MRI and four had received three courses. In three cases, it was decided to cease chemotherapy before a full six cycles were completed, as poor clinical response was being demonstrated. In these cases, MRI was, however, carried out after the final course of treatment, thus permitting a final measurement of tumour volume to be made and, thus, quantifying the relatively poor response adequately.

All five pathology variables (i.e. cancer type, grade, presence/absence of DCIS, and ERS and PRS scores) were available in 35 of the 38 eligible cases. These included 32 of the 34 valid TP_0_ MRI cases, all 22 of the valid TP_2_ MRI cases and three cases in which MRI data were unusable (see Figure 2). The age at TP_0_ for the 37 women whose data are used in this study ranged from 26 years, 6 months to 75 years, 7 months, with a median of 48 years, 6 months. The pathological cancer subtypes of these 37 cases were 19 ductal/not specified (12 with DCIS), 15 (nine) ductal and three (one) lobular. The number of women with cancers of grade one, two and three were nine, 18 and eight, respectively (cancer grade was not reported by the pathologist in two cases). Two women did not undergo surgery because of the presence of metastatic disease and one woman did not undergo surgery as post-chemotherapy core biopsy confirmed the absence of residual cancer. In the remaining cases, 17 women underwent mastectomy and 17 underwent wide local excision. The length of time between the final MRI scan and surgery ranged from 14 to 117 days, with a median of 28 days. Maximum tumour diameter was recorded in the patient's notes by both the pathologist and the MRI radiologist in 21 cases.

### Agreement between MRI and pathology measurements

Calculation of the limits of agreement between the maximum tumour diameters as measured by the pathologists and the MRI radiologist demonstrated a mean difference (MRI–pathology) of −2.1 mm with a 95% CI (1.96 × standard deviation) of ±18.6 mm. The limits of agreement were, therefore, −20.7 to 16.5 mm. A paired *t*-test of the two sets of measurements showed that the mean difference was not statistically significant (*P*=0.32), thus indicating that MRI is an unbiased estimator of true tumour size and, therefore, suitable for quantifying tumour response. The maximum diameter as measured by the MRI radiologist was also compared to the cube root of the final tumour volume, and these two variables demonstrated excellent linear correlation (*P*=0.0001; *R*^2^=0.56).

### Predicting response using individual variables

**Figure 3:**
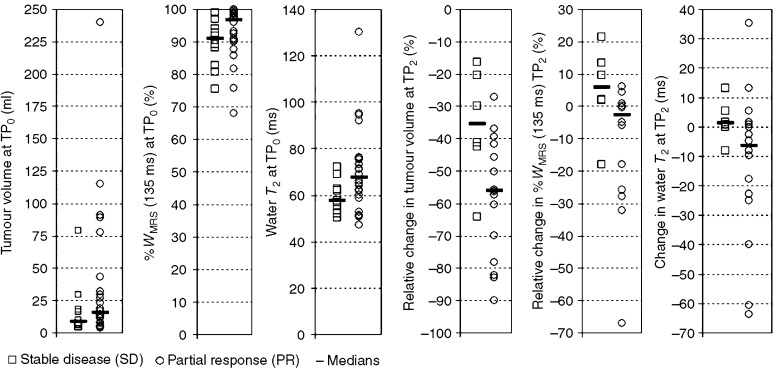
Dot plots showing the PR and SD data for six MRI variables that when correlated with a final change in tumour volume demonstrated *P*-values less than 0.07. The medians for both SD and PR subsets are also shown.

**Figure 4:**
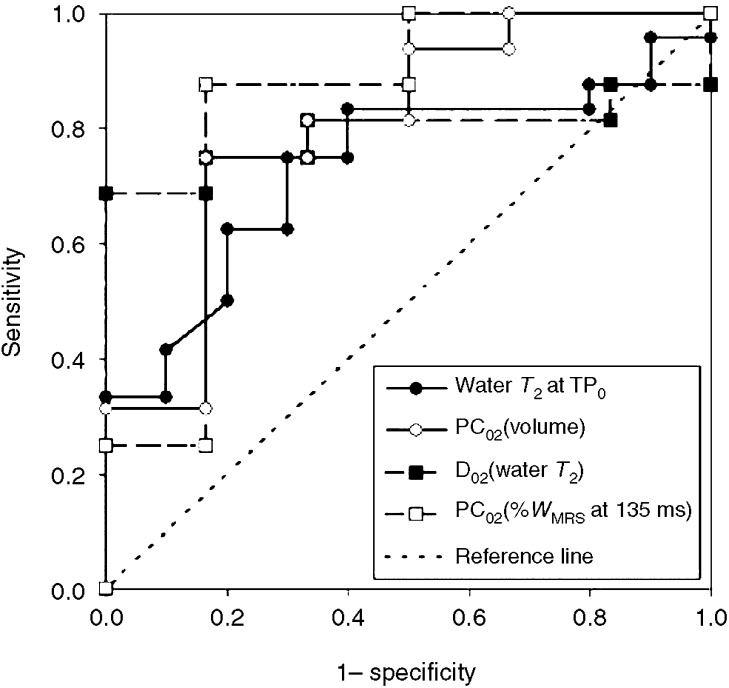
Receiver–operator characteristic curves, with PR as the positive result, for the baseline (TP_0_) water *T*_2_ (24 PR and 10 SD data) and the changes between TP_0_ and TP_2_ in spectroscopic water content measured at 135 ms, tumour volume and water *T*_2_ (16 PR and six SD data). Diagonal segments are caused by ties.

The ranges of all predictive MRI variables are presented in Table 1, and the results of correlating the predictive MRI and pathology variables with PC_0F_(*V*) are given in Table 2. Six MRI variables demonstrated *P*-values less than 0.07 and the data for these, divided into SD and PR subsets, are presented in Figure 3. These six variables along with four histopathology variables for which the *P*-values were also less than 0.07 underwent ROC curve analysis, and the results of this are also given in Table 2. The slight differences in the patterns of statistical significance between the correlation and ROC analyses can be attributed to the different variables quantifying response in the two types of tests (specifically that PC_0F_(*V*) is continuous whereas PR/SD is dichotomous). Four variables demonstrated an ROC AUC significantly larger than 0.5: water *T*_2_ at TP_0_ and changes in %*W*_MRS_ measured at 135 ms, water *T*_2_ and tumour volume between TP_0_ and TP_2_. The ROC plots for these are shown in Figure 4.

When specificity is set at 100% in Figure 4, the sensitivity for the change in water *T*_2_ at TP_2_ is 69% (11/16; 95% CI=41–89%), which suggests that this fraction of PR cases can be accurately predicted, that is, at 100% positive predictive value (PPV). Similarly, when sensitivity is set to 100%, the specificity displayed by the change in %*W*_MRS_ at TP_2_ is 50% (3/6; 95% CI=12–88%), which suggests that this fraction of SD cases can be accurately predicted, that is, at 100% negative predictive value (NPV) by this variable. The results of Table 2 also suggest that the dominant factor in driving the observed changes in %*W*_MRS_ at 135 ms (*P*=0.025) is the change in water *T*_2_ (*P*=0.006) as opposed to changes in the relative water and lipid concentrations, which would be reflected by changes in %*W*_MRS_ at 30 ms (*P*=0.788) for which the effects of *T*_2_ would be much less.

### Predicting response using a combination of variables

All variables demonstrating an ROC *P*-value less than 0.10 (Table 2) were presented as inputs to LRA modelling. Two separate analyses were conducted: one with MRI variables only (water *T*_2_ at TP_0_, PC_20_(tumour volume), *D*_20_(water *T*_2_) and PC_20_(%*W*_MRS_ at 135 ms)) and one with both MRI and pathology data (PRS score and grade). The number of cases that could be included in LRA modelling was limited to those 22 for which all of the above variables were available (16 PR and six SD). The logits for the two final models were as follows: 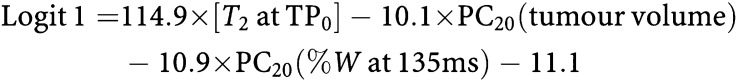


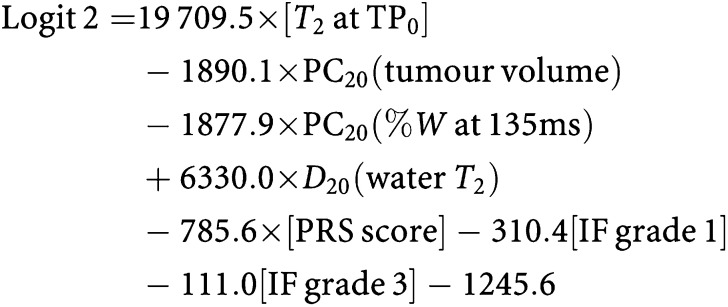


The ROC AUC values for the above logits are shown in Table 2. Both models provide an increase in prognostic accuracy over all of the individual variables, and the second model is able to classify all 22 cases accurately. Both PPV and NPV are 100% for this data set and the 95% CI lower bounds are 79% (16/16) and 54% (6/6), respectively.

## DISCUSSION

To the authors' knowledge, this paper represents the first study whereby PK parameter mapping, ADC mapping and unsuppressed proton spectroscopy (water : fat ratios) have all been investigated in the same patient examinations, thus permitting their relative abilities to predict chemotherapy response to be ascertained rigorously. The use of spectroscopic imaging at both short and long echo times represents a significant extension to the methodology of Jagannathan *et al* (1998): firstly through permitting greater post-examination control over intra-voxel partial volume effects and secondly by permitting the effects of *T*_2_ relaxation to be revealed. It was also hoped that the 30 ms TE spectra would permit investigation of the relative proportions of the 1.3 and 0.9 p.p.m. lipid peaks, along with the contributions of other, minor lipid moieties, which are more conspicuous at short TE. This analysis is not possible with quantitative chemical shift imaging (Daniel *et al*, 1998), as this technique, while having much higher spatial resolution, only provides images for water and total fat signal. It did not prove possible to resolve the separate lipid components adequately in a sufficient number of cases to permit this; therefore, chemical shift imaging may be a suitable alternative to spectroscopic imaging, permitting increased spatial resolution in future studies.

This study shows that MRS parameters provide substantial prognostic information, and slightly more than that provided by volume measurements alone, thus supporting the conclusions of Jagannathan *et al* (1998). This study also suggests that water *T*_2_ plays a dominant part in driving the observed treatment changes in water : fat ratios (at TP_2_) and it is, therefore, possible that image-based *T*_2_ mapping techniques (Liney *et al*, 1996) might provide the same prognostic information as %*W*_MRS_ but with the combined benefits of greatly increased spatial resolution, wider availability in clinical MRI centres and a reduced likelihood of technical failure in a given examination. Quantitative *T*_2_ mapping has been applied to monitoring chemotherapy in one animal tumour study, but the increases in *T*_2_ observed 3 days post-therapy were not statistically significant (Duvvuri *et al*, 2001). Significant differences were observed for *T*_1*ρ*_, however – a parameter not measured in this study because of time and technical constraints. Whether chemotherapy induces cell death via necrosis, apoptosis or a combination of the two, profound changes will certainly occur in the molecular environment of tissue water, leading to changes in its measured *T*_2_. This molecular environment is known to be complex and multicompartmental, which precludes a simple rationale for the results of this study.

Although water : fat ratios have been shown to contain substantial prognostic power, in both this and another study (Jagannathan *et al*, 1998), it is noted that a number of groups are now focusing on measurements of choline-containing compounds using water-suppressed MRS, as the choline moiety is potentially a more sensitive, and probably a more biochemically relevant marker, of cancer cell viability. Such studies have recently been carried out in breast cancer (Jagannathan *et al*, 2001; Meisamy *et al*, 2004), lymphoma and germ cell tumours (Schwarz *et al*, 2002), and glioma (Preul *et al*, 2000), where positive treatment response has been associated with reduction in the levels of total choline. Similar studies using phosphorous spectroscopy to detect changes in phosphocholine or phosphomonoesters have also been carried out in lymphoma (Arias-Mendoza *et al*, 2004) and head and neck cancer (Shukla-Dave *et al*, 2002a, 2002b). It is noteworthy that these studies attempted to predict response before treatment commencement, always a more valuable prognostic test, sparing the patients all unnecessary morbidity and time, than reliably detecting early response, which the methods presented herein achieve.

This study also demonstrates that histopathology data routinely available from pre-chemotherapy core biopsies also provide substantial prognostic power (especially so for PRS score and grade). This conclusion is supported by other laboratory studies, which have included investigation of expression of the HER2 oncogene (Penault-Llorca *et al*, 2003) and the cellular proliferation marker Ki-67 (Aas *et al*, 2003). Whereas laboratory analysis of actual tissue is inherently superior to imaging alone, it should be noted that the core biopsy technique is always prone to sampling errors, whereas imaging has the advantage of covering the whole of the lesion.

This study also demonstrates that a combination of prognostic variables, via LRA, can be used to provide a synergistic increase in prognostic accuracy, thus proving the study hypothesis. Although the perfect prognostic accuracy achieved using one of the LRA models indicates that the methods used in this study have substantial prognostic power and could be applied clinically, leading to benefits for patients, the true accuracy will be less and will need to be ascertained in larger studies. A multicentre study would also allow investigation of reproducibility. It might also be beneficial to split the data acquired in subsequent studies into separate test and validation subsets, thus allowing optimal model coefficients to be set with the former subset and their true prognostic accuracy determined through application of the model to the completely independent data in the latter.

Early, accurate detection of non-response to chemotherapy would permit an early change to second-line treatment and thus spare patients unnecessary toxicity, cost and delay of initiation of effective treatment. The cessation of treatment that would ultimately prove ineffective might also be beneficial in terms of health economics, allowing resources to be applied more efficiently. Conversely, early, accurate detection of response to chemotherapy might have positive benefits for the psychological well-being and quality-of-life of patients through giving them increased hope. The high prognostic accuracy might be bolstered by including variables that quantify lesion shape and texture, as obtained from the post-contrast three-dimensional images, in the LRA modelling (Esserman *et al*, 2001).

This study shows that ADC mapping after the second course of chemotherapy does not contribute significantly towards detecting early response. Other studies have shown that treatment-induced changes in ADC in human breast cancer and breast cancer metastasised to liver are most marked a few days after the first dose of chemotherapy (Theilmann *et al*, 2003). Therefore, our study protocol, with the predictive scans being carried out after two cycles of chemotherapy rather than one (Gibbs *et al*, 2003), was perhaps not well suited to elucidating the relative merits of ADC mapping.

In contrast to our results, other studies have indicated that PK parameters derived from DCE-MRI contain prognostic information during chemotherapy treatment in a number of locations in the human body (with reductions in *K*^trans^ being associated with positive response) as reviewed by Padhani (2002) and Knopp *et al* (2003). This apparent discrepancy regarding the efficacy of PK parameters may be due to the relatively small number of cases included in various studies, or the wide variety of DCE-MRI acquisition and PK analysis techniques used. Intra-tumour vascular heterogeneity might also be significant in some breast cancers (Hayes *et al*, 2002), leading to the possible breakdown of PK model assumptions such as the fast exchange limit within better-perfused regions (usually located at the tumour rim) (Zhou *et al*, 2004; Li *et al*, 2005). This could present a complicating factor in obtaining reliable results, an issue not addressed in this study, which utilised a whole ROI approach so as to minimise the effects of intra-scan patient motion.

Some DCE-MRI studies of breast cancer have attempted to improve the accuracy of PK modelling by imaging in the axial plane so as to allow a plasma concentration time-course to be measured in the aorta, thus permitting the calculation of an arterial input function (AIF) for the tumour. It is debatable, however, whether the benefits of including an AIF measured so distantly from the tissue of interest (i.e. one which does not allow for bolus dispersion effects) outweigh the reduction in spatial resolution and tissue coverage necessitated by axial imaging, especially when the DCE-MRI images are also used for clinical purposes. A consensus on the adoption of standardised techniques and the establishment of large, multicentre studies would, therefore, seem appropriate.

## Figures and Tables

**Table 1:** The range of values for all predictive MRI variables used in this study, as grouped by clinical time point (TP_0_ being before treatment and TP_2_ being after the second course of chemotherapy)

**Parameter (units)**	**TP_0_ (*n*=34)**	**TP_2_ (*n*=22)**
Volume (ml)	3.7–240.0	1.3–95.0
*K*^trans^ (per unit time)	1.4–18.0	0.6–8.3
*K*_ep_ (per unit time)	0.4–12.4	0.4–3.9
*V*_e_ (arbitrary)	0.6–3.5	1.2–3.1
MEF (arbitrary)	0.8–3.5	1.3–3.6
ADC (mm^2^/s)	0.3–5.9	0.9–4.3
%*W*_MRS_ at 135 ms	68–100%	30–100%
%*W*_MRS_ at 30 ms	73–100%	49–100%
Water *T*_2_ (ms)	47–130	24–104

ADC=apparent diffusion coefficient; MEF=maximum enhancement factor; MRI=magnetic resonance imaging.

**Table 2:** Statistical significance of non-parametric correlation analyses between the variables indicated and final tumour volume response PC_0F_(*V*)

	**No. of cases**	**Correlation**	**ROC curve**		
	**PR+SD**	***P* (sense)**	***P***	**AUC**	**95% CI**
*MRI data at TP_0_*					
%*W*_MRS_ at 135 ms	24+10	S: 0.034 (−)	0.14	0.66	
Water *T*_2_^[1]^	24+10	S: 0.055 (−)	0.03	0.73	0.56 to 0.91
Tumour volume	24+10	S: 0.059 (−)	0.20	0.64	
*V*_e_	24+10	S: 0.425			
*K*^trans^	24+10	S: 0.439			
%*W*_MRS_ at 30 ms	24+10	S: 0.579			
*K*_ep_	24+10	S: 0.686			
MEF	24+10	S: 0.720			
Water ADC	24+10	S: 0.808			
					
*MRI data at TP_2_*					
PC_02_(tumour volume)^[2]^	16+6	S: 0.001 (+)	0.03	0.80	0.58 to 1.00
*D*_02_(water *T*_2_)^[3]^	16+6	S: 0.006 (+)	0.04	0.79	0.60 to 0.98
PC_02_(%*W*_MRS_ at 135 ms)^[4]^	16+6	S: 0.025 (+)	0.02	0.83	0.60 to 1.00
*D*_02_(*K*^trans^)	16+6	S: 0.299			
*D*_02_(*V*_e_)	16+6	S: 0.352			
*D*_02_(*K*_ep_)	16+6	S: 0.405			
*D*_02_(MEF)	16+6	S: 0.587			
*D*_02_(water ADC)	16+6	S: 0.736			
PC_02_(%*W*_MRS_ at 30 ms)	16+6	S: 0.788			
					
*Other data*					
Age at TP_0_	26+11	S: 0.958			
Cancer grade^[5]^	24+11	S: 0.003 (−)	0.08	0.69	0.49 to 0.88
Presence of DCIS	24+11	M: 0.006 (+)	0.14	0.66	
PRS score^[6]^	24+11	S: 0.008 (+)	0.09	0.68	0.49 to 0.88
ERS score	24+11	S: 0.061 (+)	0.17	0.65	
Cancer type	24+11	K: 0.740			
					
*LRA models (variables included)*					
MRI only^[1,2,4]^	16+6		0.0032	0.92	0.79 to 1.00
MRI and histopathology^[1 to 6]^	16+6		0.0004	1.00	1.00 to 1.00

The sense of the correlations is also shown. Results of ROC curve analyses (with PR/SD as positive/negative results) are also shown for selected variables. Numbers in square brackets indicate those variables chosen as inputs to LRA modelling.

S=Spearman rank correlation; M=Mann–Whitney test; K=Kruskal–Wallis test; (+) a positive correlation with lower, or more negative values being associated with more negative values of PC_0F_(*V*), that is, PR; (−) a negative correlation with higher, or more positive values being associated with more negative values of PC_0F_(*V*), that is, PR.

AUC=area under the curve; ADC=apparent diffusion coefficient; DCIS=ductal carcinoma *in situ*; ERS=oestrogen receptor status; LRA=logistic regression analysis; MEF=maximum enhancement factor; MRI=magnetic resonance imaging; PR=partial response; PRS=progesterone receptor status; ROC=receiver–operator characteristic; SD=stable disease.
