# Neoadjuvant chemotherapy in breast cancer: early response prediction with quantitative MR imaging and spectroscopy

**DOI:** 10.1038/sj.bjc.6603165

**Published:** 2006-05-16

**Authors:** D J Manton, A Chaturvedi, A Hubbard, M J Lind, M Lowry, A Maraveyas, M D Pickles, D J Tozer, L W Turnbull

**Correction to:** *British Journal of Cancer* (2006) **94**, 427–435. doi:10.1038/sj.bjc.6602948

Owing to an author error, the units quoted for the apparent diffusion coefficient (ADC) data in the above paper are wrong.

The data themselves are correct, but units of mm^2^/s were quoted, rather than 10^−3^mm^2^/s.

The authors would like to point out that, although they do not think that this represents a major flaw – given that the paper was concerned with the relative degree of change in ADC values during treatment for partial response and stable disease cases, not the absolute ADC values – they would, nevertheless, prefer to note its correction.

